# Reduced Surface Expression of Epithelial E-Cadherin Evoked by Interferon-Gamma Is Fyn Kinase-Dependent

**DOI:** 10.1371/journal.pone.0038441

**Published:** 2012-06-08

**Authors:** David Smyth, Gabriella Leung, Maria Fernando, Derek M. McKay

**Affiliations:** Gastrointestinal Research Group, Department of Physiology & Pharmacology, Calvin, Phoebe and Joan Snyder Institute for Chronic Diseases, University of Calgary, Calgary, Alberta, Canada; Ottawa Hospital Research Institute, Canada

## Abstract

Interferon gamma (IFNγ) is an important regulatory cytokine that can exert a pro-inflammatory effect in the gut, where it has been shown to increase epithelial permeability via disruption of the tight junctions. Here we investigated the potential for IFNγ to regulate the adherens junction protein E-cadherin, an important mediator of normal epithelial tissue function, using the model T84 human colonic epithelial cell line. IFNγ (10 ng/ml) stimulated increased internalization of E-cadherin as assessed by immunofluorescence microscopy; internalization was reversed when cells were treated with PP1 (125 nM), a Src kinase-selective inhibitor. Immunoprecipitation studies demonstrated loss of E-cadherin from membrane fractions following IFNγ treatment and a corresponding increase in cytosolic E-cadherin and its binding partners, p120-catenin and beta-catenin: effects that were Src-kinase dependent. E-cadherin and p120-catenin phosphorylation was increased by IFNγ treatment and siRNA studies showed this was dependent upon the Src-kinase isoform Fyn. E-cadherin ubiquitinylation and subsequent proteasomal degradation stimulated by IFNγ was found to be dependent upon Fyn and the E-cadherin-selective ubiquitin ligase, Hakai. Use of Fyn and Hakai siRNA inhibited the internalization of E-cadherin as shown by immunoblotting and confocal fluorescence microscopy. Finally, IFNγ treatment resulted in a more fragile T84 cell monolayer with increased cell detachment in response to physical stress, which was prevented by PP1 and siRNA targeting Fyn or Hakai. Collectively, these results demonstrate a Fyn kinase-dependent mechanism through which IFNγ regulates E-cadherin stability and suggest a novel mechanism of disruption of epithelial cell contact, which could contribute to perturbed epithelial barrier function.

## Introduction

The integrity of the intestinal epithelial monolayer constitutes an important regulated barrier that controls access of the gut microflora, an abundant population of commensal and potentially pathogenic microbes, to the mucosa and thus is a key modulator of immune-mediated inflammatory activity within the intestinal submucosa [1]. Furthermore, increases in enteric epithelial permeability often parallel the onset of inflammatory disease and also potentially colorectal cancer [2]. As the association between microbial-driven inflammation and cancer becomes more apparent, a greater awareness of mechanisms of altered gut epithelial function during inflammatory responses may lead to additional or improved treatment strategies.

Interferon-gamma (IFNγ) is a key inflammatory cytokine primarily secreted by T cells and natural killer (NK) cells that has a well-described role during intestinal inflammation [3], [4]. IFNγ stimulates increased intestinal epithelial permeability by reducing tight junction stability (paracellular permeability) [5], and our work, and that of others, has shown that IFNγ signalling through phosphatidylinositol 3-kinase (PI3K) and the Src-kinase family member, Fyn, promotes increased epithelial uptake of commensal bacteria and macromolecules in *in vitro* model systems [6]–[8]. Thus, IFNγ may serve to exacerbate inflammatory responses via its effects on the epithelial barrier.

Src kinase activity has been widely-associated with epithelial dysfunction. Src kinases have been linked to epithelial to mesenchymal transition (EMT) in response to growth factors or oxidative stress [9], [10]. c-Src-mediated epithelial cell scattering has been suggested as an important step during acquisition of a transformed phenotype [11], and the Src-family Fyn kinase has recently been identified as a potential catalyst for the development of prostate cancer [12], [13]. However, extensive studies of Fyn kinase participation in the intestinal epithelial response to IFNγ have not been conducted.

A key mediator of the stability of the intestinal epithelium is the adherens junction. E-cadherin is a critical intercellular junctional protein that is maintained at the cell surface by interactions with p120-catenin, beta-catenin and additional proteins mediating adhesion to the actin cytoskeleton [14]. Loss of E-cadherin function is associated with development of chronic inflammatory diseases including Crohn’s disease [15]. E-cadherin is also considered a tumour suppressor, as loss of E-cadherin expression or activity is highly correlative to the onset of epithelial-derived cancers [16]. Fyn kinase has been reported to induce E-cadherin internalization following epithelial cell exposure to acidic pH or growth factors *in vitro* [17], [18], and p120-catenin, whose key function is to bind and stabilize E-cadherin, is known to be a Fyn kinase substrate [19]. Thus, we sought to determine whether IFNγ-stimulated Fyn kinase activity affected epithelial adherens junction stability, specifically the expression and location of E-cadherin.

Here we show that IFNγ stimulates increased internalization of E-cadherin in the human colonic T84 epithelial cell line. E-cadherin internalization was reduced by treatment with the Src inhibitor PP1 and siRNA targeting Fyn, and by siRNA targeting Hakai, which has been characterized as a Src-dependent, E-cadherin-specific ubiquitin ligase. Blockade of Fyn or Hakai reduced the fragility of cell-cell adhesion in IFNγ-treated plastic-grown T84 cell monolayers. The data illustrate that an adherens junction-destabilizing pathway involving Fyn kinase and Hakai can be activated by IFNγ in T84 epithelia, and we speculate that this could be relevant to epithelial cell-cell adhesion and communication, and potentially enteric inflammatory disease.

## Materials and Methods

### Reagents and Antibodies

Cell culture supplements and pharmacologic inhibitors, were purchased from Sigma-Aldrich (Oakville, Ontario, Canada) unless otherwise indicated. The Src inhibitor PP1 was purchased from Biomol (Enzo Life Sciences, Plymouth Meeting, PA, USA). Recombinant human IFNγ was from Ebioscience Inc. (San Diego, CA, USA). Mouse anti-E-cadherin and mouse anti-beta-catenin antibodies were purchased from BD Transduction Labs (Mississauga, ON, Canada). Mouse anti-p120 catenin and mouse anti-phosphotyrosine (clone 4G10) antibodies were purchased from Upstate/Millipore (Billerica, MA, USA). Rabbit anti-occludin was purchased from Zymed/Invitrogen (Carlsbad, CA). Rabbit anti-zonula occludens (ZO-1) antibody was purchased from Invitrogen. Goat anti-actin antibody, rabbit anti-CBLL/Hakai, mouse anti-Fyn antibody and HRP-conjugated secondary antibodies were from Santa Cruz Biotech (Santa Cruz, CA, USA). AlexaFluor goat anti-mouse 488 and goat anti-rabbit 594 fluorescent secondary antibodies were purchased from Molecular Probes/Invitrogen (Carlsbad, CA, USA).

### Cell Culture

The immortalized human colon-derived T84 epithelial cell line (ATCC, Manassas, VA, USA) was cultured at 37°C/5% CO_2_ in 1∶1 Dulbecco’s modified Eagle’s Medium/Ham’s F-12 medium supplemented with 2% (vol./vol.) penicillin-streptomycin, 1.5% HEPES, 5% NaHCO_3_, 1% L-glutamine, 1% sodium pyruvate (all from Invitrogen, Burlington, ON, Canada) and 10% fetal bovine serum (PAA Laboratories, VWR International, Edmonton, AB, Canada). All cytokine stimulations with IFNγ were conducted using 10 ng/ml recombinant cytokine (equivalent to 250 biological units of activity/ml cell culture medium).

### Transient Transfection of T84 Cells with Small Interfering (si) RNA

siRNAs targeting Fyn and Hakai were created using the Stealth siRNA oligomer design platform (Invitrogen). Target oligomer sequences used in this study are as follows:

Fyn 5′-GAGCGACAGCTATTGTCCTTTGGAA. Hakai 5′ -CAACATGTGCCACATGAGCA CTATA. The control siRNA sequence used was 5′-GAGACATCGTTACTGTTCGGAA. Transfections were performed as previously described [8]. Briefly, 20 pM of siRNA in Lipofectamine 2000/Opti-MEM (500 µg/ml) (Invitrogen) was added to suspension cultures of T84 cells (1×10^6^/ml) in antibiotic-free FBS-containing culture medium. The cells were then either seeded onto filter supports or 12-well culture dishes and following an overnight incubation, adherent cells were washed and transferred to antibiotic-containing culture medium.

**Figure 1:**
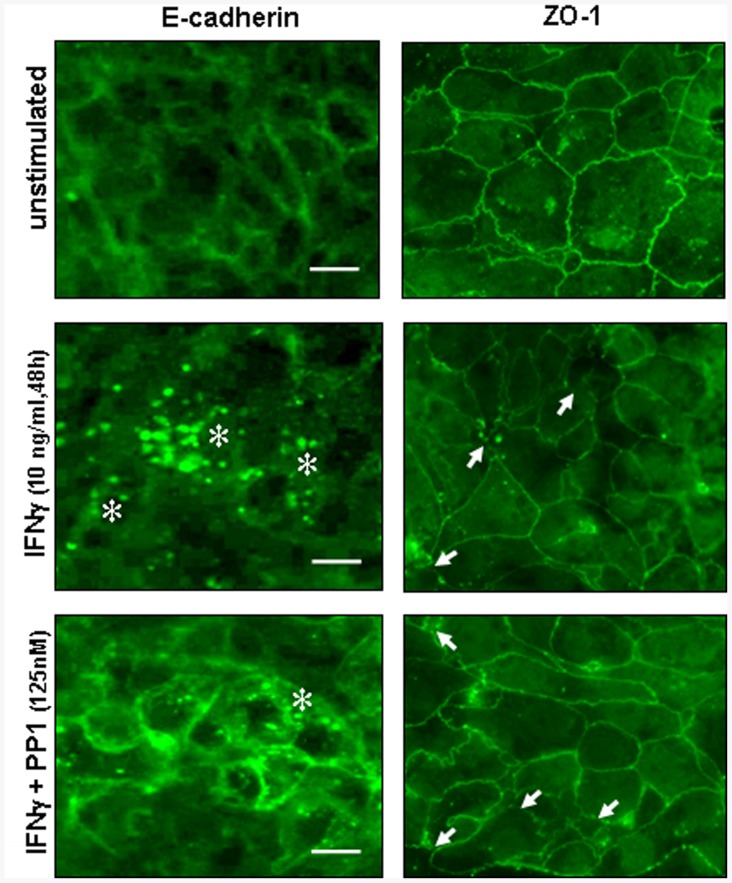
The Src inhibitor PP1 blocks IFNγ -stimulated E-cadherin internalization but does not affect the focal loss of zonula occludens-1 (ZO-1). T84 cells were grown on glass coverslips and stimulated with IFNγ (10 ng/ml, 48 h)±the Src inhibitor PP1 (125 nM). Left panels: immunofluorescence microscopy of epithelial monolayers probed with a C-terminal-specific anti-E-cadherin antibody showed IFNγ caused increased E-cadherin internalization (*), which was inhibited by PP1 co-treatment, whereas in contrast (right panels) the focal discontinuities in the distribution of ZO-1 induced by IFNγ (arrows) was unaffected by PP1 co-treatment. Images collected are representative of two independent experiments each using replicate epithelial cell monolayers per treatment, the junctional pattern with E-cadherin reflects the more diffuse adherens junction which is in contrast to the more localized tight junction and associated plaque proteins (i.e. ZO-1). Scale bar = 10 µm.

### Immunoprecipitation and Immunoblotting

One million T84 cells were seeded onto 12 mm^2^ permeable filter supports (Greiner Bio-One) in 12-well plates and cultured until confluent as assessed by phase-contrast microscopy and electrical confluence (transepithelial resistance (TER)≥1000 Ω**.**mm^2^ as measured by voltmeter and paired electrodes (Millipore) was accepted as an electrically confluent monolayer). As indicated, cell lysates were prepared following two washes with ice-cold phosphate-buffered saline (PBS). For low-salt, detergent-free lysate generation, cells were scraped into hypotonic Buffer A as described in [20] supplemented with protease and phosphatase inhibitors (Complete® protease inhibitor cocktail (Roche/Mannheim), 1 mM sodium orthovanadate, 1 mM sodium fluoride). Lysates were incubated with gentle agitation at 4°C for 30 min, centrifuged at 10,000×g and supernatants were collected and stored at -80°C. Protein concentrations were determined by Bradford assay (Bio-Rad, Hercules, CA, USA). For isolation of membrane or cytoskeletal components, T84 monolayers were lysed in 1% sodium dodecyl sulphate (SDS)/PBS supplemented with protease inhibitors as above. Isolation of equivalent quantities of cell material was assured by adjustment to Triton X-100 protein lysate concentrations of identically plated cell cultures. For immunoprecipitation experiments, monolayers were extracted using radioactive immunoprecipitation assay (RIPA) buffer (100 mM NaCl, 24 mM Tris-Cl, 1% (vol./vol.) NP-40, 0.5% sodium deoxycholate, 0.1% SDS) supplemented with Complete® protease inhibitor cocktail, sodium orthovanadate and sodium fluoride). Four hundred µg of clarified cell lysates were incubated in 2 µg/ml anti-E-cadherin antibody overnight at 4°C with gentle agitation. Immune complexes were isolated by incubation with EZ-link protein A-agarose beads (Sigma) at 4°C for 90 mins, followed by two washes in RIPA buffer and one wash with PBS. Immune complexes were eluted with 2X Laemmli buffer and set aside for immunoblotting. Protein lysate immunoblotting was performed by addition of 20 µg of lysates to Laemmli buffer, which were subsequently boiled and resolved on 8% SDS-PAGE. Separated proteins were blotted to Immobilon nitrocellulose membranes (Millipore), and blots were blocked at room temperature for 1 h in 5% non-fat milk/wash buffer (0.15% Tween-20/Tris-buffered saline (TBS/T)). Primary antibodies (see Results) were incubated in 1% bovine serum albumin/TBS/T (for phosphoprotein analysis) or 5% non-fat milk/TBS/T (total proteins) overnight at 4°C with gentle rocking. Blots were washed three times in TBS/T and species-appropriate, HRP-conjugated secondary antibodies were applied with gentle rocking for 1 h at room temperature. Blots were washed, subjected to chemiluminescence (Western Lightning® PLUS, PerkinElmer, Waltham, MA, USA) and subsequently exposed to Kodak XB-1 film (Eastman Kodak, Rochester, NY, USA).

Densitometric quantification of phospho-E-cadherin and ubiquitinylated-E-cadherin chemiluminescence was performed by analysis of 16-bit JPEG blot images with Image J (version 1.45, NIH open access software, W. Rasband). Measurement was conducted on three replicate experimental immunoblots. Levels of phospho-E-cadherin or ubiquitinylated-E-cadherin were normalized to total E-cadherin immunoprecipitated per sample, and measurements presented as relative ratios compared to non-stimulated control (scored as a value of ‘1’). As controls, input lysate levels of E-cadherin were assessed and actin levels from unbound, or flow-through lysate, were also verified.

### Immunofluorescence Microscopy

T84 cells were plated (3×10^5^ cells/ml) onto glass coverslips in 12 well culture plates and grown to 70% confluent, at which time IFNγ±PP1 (125 nM) was added for 48 h. Coverslips were washed three times in 4°C PBS, fixed in 4% paraformaldehyde (PFA), washed three times in PBS and then blocked with 10% goat serum for 1 h at room temperature. Monolayers were then incubated for 24 h at 4°C with mouse anti-E-cadherin (1∶350) or anti-ZO-1 (1∶100) antibody in PBS containing 10% goat serum and 0.1% Tween. Following three washes with 1X PBS, monolayers were incubated for 1 h at room temperature with AlexaFluor 488 goat anti-mouse secondary antibody (1∶500). DAPI (1∶500) was added for 1 min followed by 2 washes with 1X PBS, and coverslips were mounted onto slides with Fluorosave (Calbiochem), allowed to dry and stored in dark at 4°C. For visualization, slides were analyzed using an Olympus 4100BX epifluorescence microscope (Olympus) using the 40X objective lens: regions of monolayer were randomly selected based on DAPI-identification of nuclei and then specific immunofluorescence observed and images captured of that area.

#### Confocal laser scanning microscopy

For some experiments, T84 cells were transfected with control, Fyn or Hakai siRNA and seeded onto 6 mm^2^ filter supports at 1×10^5^ cells/ml. Following 72 h, cells were either left untreated or stimulated with IFNγ for 48 h, and then washed and fixed as above. Subsequently, cells were stained with anti-E-cadherin antibody or anti-occludin antibody (1∶350) then with appropriate AlexaFluor secondary antibodies (1∶500), mounted and images captured on an Olympus FV1000 confocal scanning fluorescent microscope (40X objective). Images were collected and analyzed using FV10-ASW2.1 imaging software (Olympus). As described above, cell viability and localization were verified by nuclear DAPI staining. Determination of plane depth for analysis of occludin and E-cadherin was carried out using occludin-immunoreactivity as the indicator of the apical aspect of the epithelial layer. For each captured image E-cadherin was imaged 0.2 µm deeper than the z-plane slice selected for occludin localization.

### Cell Detachment Assays

T84 cells were seeded on 12 mm^2^ permeable filter supports as described previously and grown to confluence and indicated by TER≥1000 Ω**.**cm^2^. Where indicated, cells were transfected with control, Fyn or Hakai targeted siRNA. Control siRNA-transfected cells were either left untreated or treated with IFNγ±125 nM PP1. Fyn and Hakai siRNA-treated T84 cells were also stimulated with IFNγ. Following 48 h cytokine treatment, TER was measured, the cells washed twice with 1 ml of 1X PBS and incubated for 15 minutes at 37°C in 500 µl serum-free PBS. Subsequently, epithelial monolayers were rinsed gently by pipeting with PBS (ten passes per monolayer) and detached cells were collected into 1.5 ml Eppendorf tubes, placed on ice, and centrifuged for 2 min at 3000×g. Cell number was assessed in two ways: (1) cells were re-suspended in 100 µl PBS and were deposited onto slides (50 µl/preparation) by Cytospin, stained with Cresyl violet, and then counted at 20× magnification on a bright field inverted microscope. Counts were made for each filter-grown epithelial monolayer, with a minimum of triplicate filters used per treatment condition; (2) cell number was approximated by quantification of total protein from the lysis of collected detached cells (using 100 µl RIPA buffer/filter collected) using the Bradford assay. Protein was measured according to a standard concentration curve using bovine serum albumin and results were plotted graphically as µg of detached cell protein isolated per filter; cell detachment was plotted as a percentage of detachment relative to non-stimulated control (which was assigned a percentage value of 100).

### Statistical Analysis

Quantitative data are presented as mean±standard error of the mean (SEM), with n values given as the number of epithelial observations from replicate experiments. Single group comparisons were performed using Student’s t test and multiple group statistical analysis was by a one-way analysis of variance (ANOVA) followed by pair-wise post-hoc statistics. For cell detachment studies, sample sizes were: non-stimulated (control), n = 5; IFNγ, n = 6; IFNγ+PP1, n = 5; IFNγ+Fyn siRNA, n = 5; IFNγ+Hakai, n = 4. In all analyses a p<0.05 was accepted as a level of statistically significant difference.

**Figure 2:**
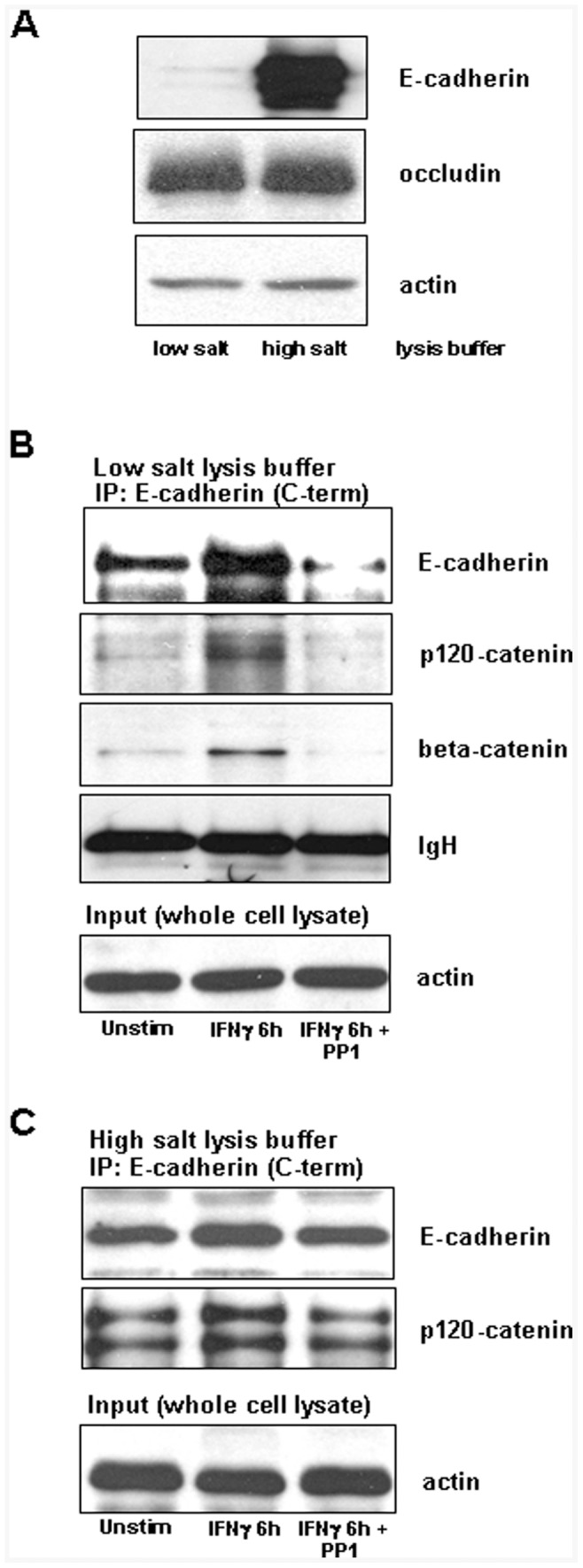
Increased solubilization of E-cadherin, p120-catenin and beta-catenin by IFNγ is Src-dependent. A) Representative immunoblot showing differential solubility of E-cadherin, but not occludin, in low-salt buffer compared to high salt-1% SDS lysis buffer. B) E-cadherin is associated with p120-catenin and beta-catenin and complex solubility is increased by IFNγ (10 ng/ml, 6 h) treatment as shown by SDS-PAGE of detergent-free (low-salt) lysates immunoprecipitated with anti-E-cadherin antibody. Inclusion of PP1 (125 nM) inhibits increased solubilization of E-cadherin/p120-catenin/beta-catenin caused by IFNγ. Data shown are representative of three independent experiments. C) Immunoprecipitation of E-cadherin and p120-catenin following lysis in high-salt-containing buffer shows only marginal increase in expression in the IFNγ treated epithelia. IgH and actin are included as loading controls.

**Figure 3:**
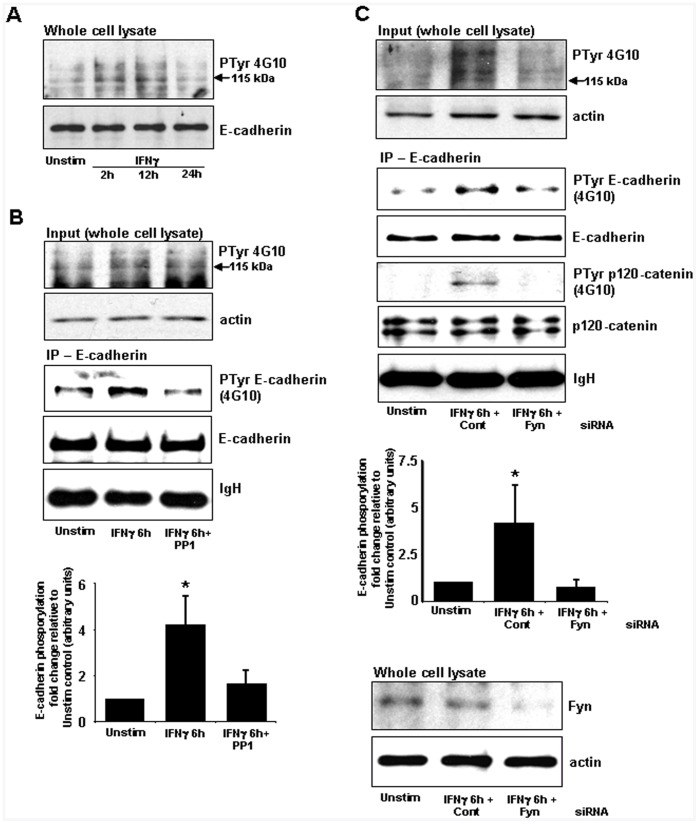
IFNγ-stimulated phosphorylation of E-cadherin and p120-catenin is reduced by PP1 and Fyn kinase siRNA. A) Time course analysis reveals a generalized increase in tyrosine phosphorylation in T84 whole cell lysates treated with IFNγ (10 ng/ml). B) Tyrosine phosphorylation of E-cadherin following IFNγ stimulation (6 h) was reduced by PP1 (125 nM) as demonstrated in a representative immunoblot of E-cadherin-immunoprecipitated T84 cell lysates and quantified by densitometric assessment conducted on the result of three representative experiments. C) Increased E-cadherin and p120 catenin tyrosine phosphorylation evoked by IFNγ was reduced in cells in which Fyn expression (lowest panels) had been knocked-down by siRNA. Actin and IgH are included as loading controls. Graph depicts densitometry analysis of phospho-E-cadherin immunoblots from three experiments (mean±SEM; *, p<0.05 compared to control (Unstim) and IFNγ+Fyn siRNA treated epithelia).

## Results

### E-cadherin Internalization is Increased Following IFNγ Treatment in a Src-dependent Manner

Previous research indicates that IFNγ is a potent modulator of intestinal tight junction form; however, relatively little attention has focused on the role of IFNγ in the regulation of the adherens junction. IFNγ has been reported to reduce surface expression of E-cadherin in intestinal epithelial cells [21], but the intracellular mechanism(s) responsible for this was not determined. Given our data identifying a key role for the Src kinase Fyn in IFNγ-evoked increases in epithelial barrier function [8], we sought to determine if Src activity was required for IFNγ-evoked changes in E-cadherin expression and localization in T84 epithelia. Initial studies used epifluorescence microscopy to obtain a view of the global impact of IFNγ treatment on E-cadherin expression. Forty-eight hours after IFNγ treatment (10 ng/ml), there was a marked accumulation of E-cadherin in cytosolic punctate structures and loss from the peri-junctional area, that was reduced by co-treatment with the pan-Src inhibitor PP1 (Figure 1). As a comparison with tight junction structure, a 48 h treatment with IFNγ resulted in the expected focal discontinuities in ZO-1 [21], that was unaffected by PP1 co-treatment (Figure 1).

### IFNγ Treatment Stimulates Increased Adherens Junction Protein Accumulation in a Soluble Cytoplasmic Isolate

E-cadherin typically localizes to a highly insoluble cellular fraction enriched with cytoskeletal components. We hypothesized that IFNγ stimulation would provoke a release of E-cadherin and interacting proteins from the cell membrane to the cytosol, where it would be extractable under less stringent isolation conditions. Lysis of equivalent-density cell cultures in either detergent-free, hypotonic conditions (termed low-salt buffer) or 1% SDS buffer (a highly disruptive, anionic detergent buffer) indicated that the majority of E-cadherin remained insoluble in non-stimulated T84 cells (Figure 2A). Conversely, equivalent amounts of the tight junction protein occludin were identified by immunoblotting of extracts retrieved by the low-salt and SDS buffers. Analysis of T84 cell extracts obtained with the low-salt buffer revealed increased E-cadherin after 6 h of IFNγ treatment, indicative of movement out of the membrane and into the cytosol (Figure 2B). Similarly, co-immunoprecipitation experiments employing a monoclonal antibody raised against a C-terminal E-cadherin epitope revealed increased amounts of detectable p120-catenin and beta-catenin, two binding partners of the C-terminal domain of E-cadherin, in the low-salt extract: suggestive of the E-cadherin/p120 catenin/beta-catenin disengagement from the cell membrane. No detectable E-cadherin, p120 catenin or beta-catenin could be recovered from control, non-immune IgG immunoprecipitated samples (data not shown). Inhibition of Src kinases with PP1 (125 nM, a concentration that targets Fyn) reduced the solubility of all three proteins following IFNγ stimulation. In contrast to low-salt extracted conditions, immunoprecipitation of high-salt lysis buffer-extracted E-cadherin showed a minor and insignificant increase in protein levels of both E-cadherin and p120-catenin following IFNγ treatment (Figure 2C).

**Figure 4:**
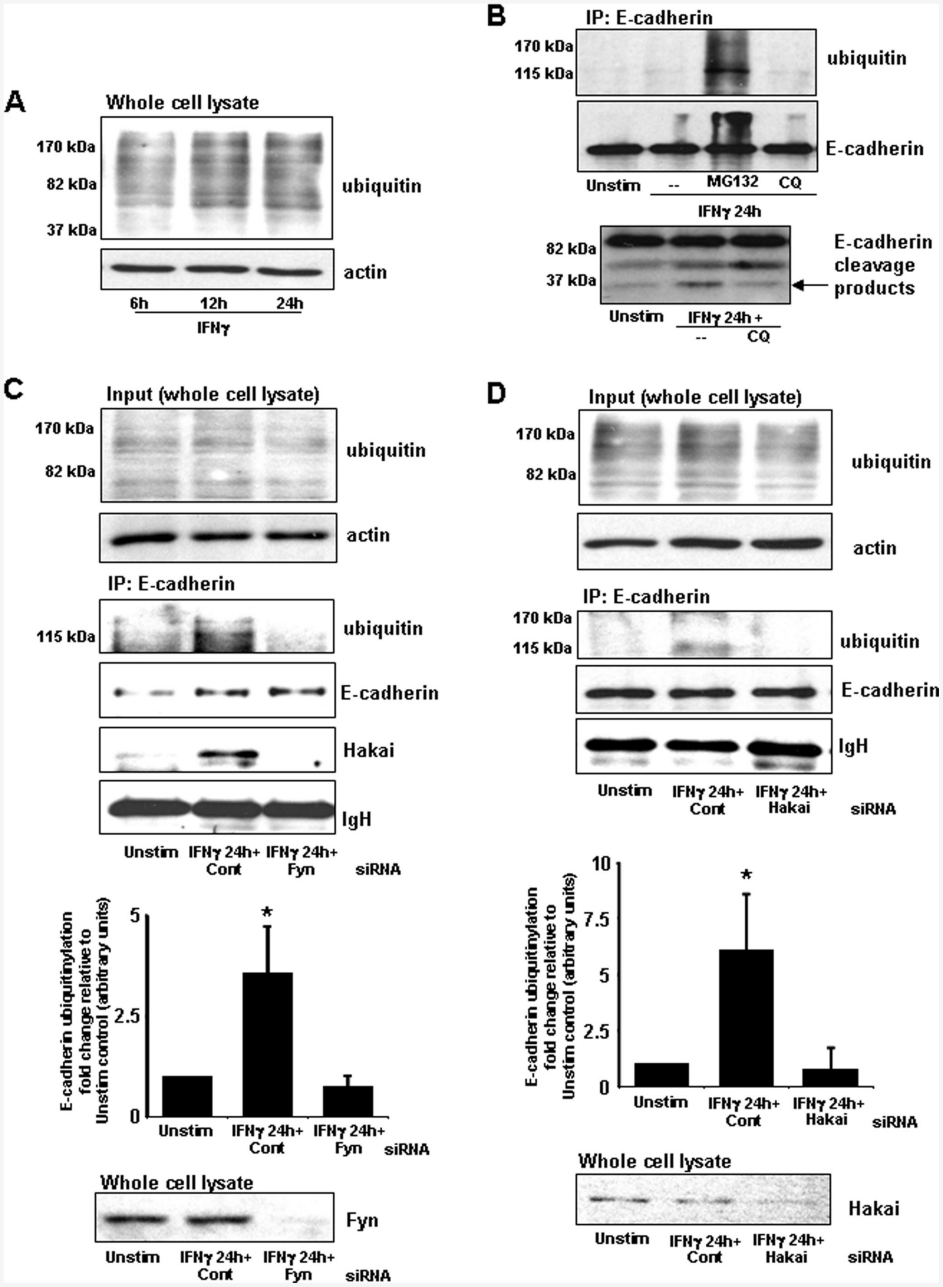
Ubiquitinylation of E-cadherin stimulated by IFNγ is inhibited by siRNA targeting Fyn kinase and Hakai. A) Representative immunoblot showing a time-dependent increase in total ubiquitinylation induced in T84 epithelial cells treated with IFNγ (10 ng/ml). B) Upper panels demonstrate that treatment with the proteosome inhibitor, MG132 (500 nM), but not chloroquine (CQ, 5 mM; reduces lysosomal acidification) prevents the IFNγ-evoked degradation of E-cadherin as shown by the presence of ubiquitinylated E-cadherin. Lower panels show cytoplasmic cleavage products of E-cadherin. Levels of the 30 kDa lysosomal fragment (arrowhead) were increased following IFNγ treatment but were reduced in IFNγ+chloroquine-treated samples. C) Immunoprecipitated E-cadherin demonstrated ubiquitinylation following IFNγ stimulation which was reduced following Fyn siRNA. E3 ubiquitin ligase Hakai is co-immunoprecipitated with E-cadherin following IFNγ stimulation but is reduced in Fyn siRNA treated epithelia. Densitometric analysis (performed in triplicate) is shown below a representative immunoblot. Whole lysates (input) demonstrate knockdown of Fyn by siRNA. D) Hakai siRNA inhibits E-cadherin ubiquitinylation; whole cell lysates indicate Hakai knockdown achieved by siRNA. Densitometry is shown below a representative immunoblot (mean±SEM; *, p<0.05 compared to controls (Unstim) and IFNγ+Fyn (or Hakai) siRNA treated epithelia; actin and IgH are included as loading controls).

**Figure 5:**
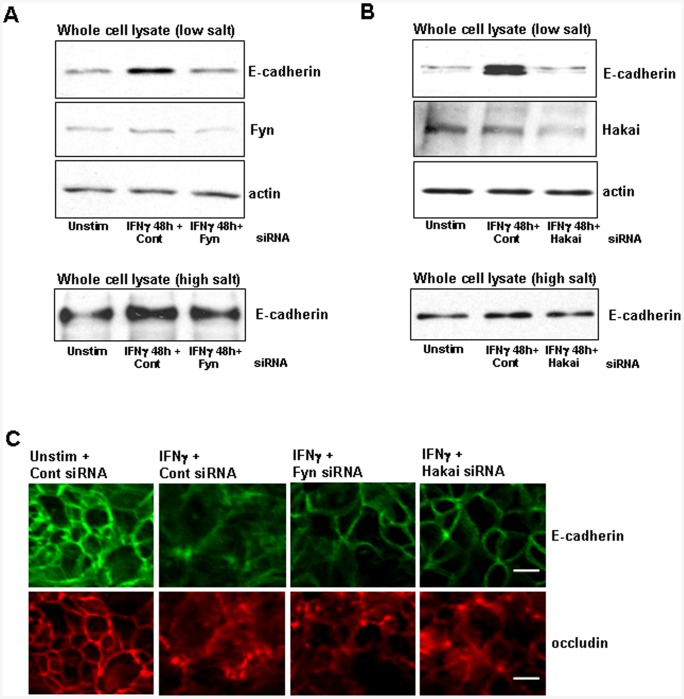
Hakai siRNA inhibits IFNγ-stimulated internalization of E-cadherin. Low salt, detergent-free lysis of MG132 (500 nM)-pretreated T84 cells reveal that IFNγ-stimulated (10 ng/ml, 48 h) internalization of E-cadherin is reduced in presence of A) Fyn and B) Hakai siRNA. High-salt lysates do not demonstrate as dramatic an increase in solubility relative to low-salt lysis (lower panels). Data are representative of two experiments for each lysis preparation. C) Confocal photomicrographs of immunofluorescent detection of E-cadherin and occludin localization. IFNγ treatment disrupts the pattern of E-cadherin (adherens junction) and occludin (tight junction) distribution, and treatment with Fyn kinase and Hakai siRNA partially abrogates the displacement of E-cadherin but not occludin from the peri-junctional region. Data shown are representative of three experiments for Fyn, two experiments for Hakai. Scale bar = 10 µm.

### IFNγ-stimulated Tyrosine Phosphorylation of E-cadherin and p120 Catenin is Fyn-dependent

We subsequently focused on identifying potential mechanisms responsible for the IFNγ-evoked increase in E-cadherin solubility (i.e. dissociation from the membrane). Studies of v-Src transformed cell lines suggest that increased tyrosine phosphorylation of E-cadherin results in its dissociation from the cell surface; while p120-catenin phosphorylation has been shown to affect its ability to stabilize membrane-bound E-cadherin [22]. Anticipating that an increase in phosphorylation of total E-cadherin would reflect the increased amounts of solubilised E-cadherin (Figures 1 and 2), epithelial cell lysis was conducted using RIPA buffer followed by SDS-PAGE and immunoblotting. IFNγ treatment evoked a general increase in tyrosine phosphorylation in T84 cells (Figure 3A). Following 6 h of IFNγ stimulation, there was increased tyrosine phosphorylation of E-cadherin, as demonstrated by immunoblotting of immunoprecipitated E-cadherin with the anti-phosphotyrosine antibody 4G10. Consistent with our observation of altered E-cadherin solubility, the Src inhibitor PP1 treatment strongly inhibited IFNγ-stimulated E-cadherin phosphorylation (Figure 3B). We had previously reported that use of PP1 at low concentrations could exclude potential off-target inhibition of tyrosine phosphorylation by receptor tyrosine kinases such as EGFR or by inhibition of Janus kinases, and we showed that Fyn was a major Src family member stimulated by IFNγ in T84 cells [8]. Therefore, the effects of Fyn siRNA upon E-cadherin phosphorylation were examined. Consistent with the data from PP1, immunoprecipitates of E-cadherin demonstrated reduced tyrosine phosphorylation following IFNγ treatment in the presence of Fyn siRNA as compared to control siRNA-treated lysates (Figure 3C). Additionally, IFNγ-stimulated p120-catenin phosphorylation was also reduced in T84 cells treated with Fyn siRNA (Figure 3C). Consistent with the results presented in Figure 2B and C, control (non-immune) mouse IgG antisera did not immunoprecipitate E-cadherin, validating immunoblot experiments (data not shown).

### E-cadherin Ubiquitinylation is Stimulated by IFNγ and is Dependent upon Fyn and Hakai

Next we wanted to identify factors that may promote the internalization of tyrosine phosphorylated E-cadherin. Research examining the mechanisms of epithelial cell infection by the enteric pathogen *Listeria monocytogenes* has indicated that the internalization of E-cadherin may occur in part via ubiquitin-dependent mechanisms. Here, we found increased ubiquitinylation in whole cell lysates at 6–24 h post-IFNγ treatment (Figure 4A). Immunoprecipitated E-cadherin from T84 cells treated 24 h previously with IFNγ was ubiquitinylated (Ub), but this was only apparent in samples from epithelia in which proteasome activity was inhibited by MG132 (Figure 4B). Conversely, treatment of T84 cells with chloroquine, an inhibitor of lysosomal activity, did not prevent the degradation of Ub-E-cadherin induced by IFNγ. As an indicator of chloroquine activity, we observed a decreased amount of lysosomal-associated E-cadherin fragmentation. Cleavage of lysosomal-targeted E-cadherin is evidenced by a marked E-cadherin C-terminal 30 kDa peptide [23]. The lower panel of Figure 4B shows the soluble cytoplasmic fragments of E-cadherin, including an approximately 85 kDa peptide fragment released by cleavage of the extracellular domain and a 30 kDa fragment consistent with the size of the lysosomal-generated E-cadherin peptide. IFNγ-stimulated T84 cell lysates demonstrated greater levels of 30 kDa E-cadherin, but less of the protein fragment was present in cell lysates from IFNγ+chloroquine treated cultures (Figure 4B). Additionally, only Ub-E-cadherin, not ‘unmodified’ E-cadherin levels, were markedly reduced by MG132. We then focused on candidate ubiquitinylation mechanisms that could direct E-cadherin to the proteasome for degradation. Hakai is a phosphorylation-dependent RING-type E3 ligase with a reported specificity for E-cadherin [24]. Hakai-mediated ubiquitinylation of E-cadherin is Src-dependent and promotes the proteasomal degradation of E-cadherin [24], [25]. Thus, we sought to determine whether IFNγ-stimulated E-cadherin phosphorylation by Fyn was required for Hakai mediated ubiquitinylation of E-cadherin. The upper panels of Figure 4C presented ubiquitinylation immunoblotting from RIPA-isolated T84 whole cell lysates. Irrespective of treatment, whole cell ubiquitin levels were only slightly modulated. However, from E-cadherin immunoprecipitates (shown in the lower panels) we observed that levels of ubiquitinylated E-cadherin following 24 h IFNγ exposure were significantly increased. In contrast, IFNγ-stimulated T84 cells treated with Fyn siRNA demonstrated a significant reduction of ubiquitinylation, consistent with a requirement for tyrosine phosphorylation of E-cadherin prior to ubiquitinylation (Figure 4C). Hakai co-immunoprecipitated with E-cadherin following IFNγ treatment, and this association was reduced in Fyn siRNA-treated epithelia. We then assessed the effects of Hakai siRNA upon ubiquitinylation of E-cadherin (Figure 4D). Similarly to Fyn siRNA treatment, whole cell lysate immunoblots (upper panels) showed marginal changes to overall ubiquitinylation levels, but immunoprecipitation of E-cadherin (lower panels) indicated an IFNγ-stimulated increase in Ub-E-cadherin. Hakai siRNA-treated T84 epithelia displayed significantly reduced levels of Ub-E-cadherin following IFNγ treatment.

### Fyn- and Hakai-specific siRNA Reduce IFNγ-stimulated E-cadherin Internalization

Initial studies indicated that IFNγ treatment of T84 epithelia resulted in a relatively rapid internalization of E-cadherin (Figure 2B), but what of the effects, if any, in a longer time-frame? To address this, we used the proteasome inhibitor, MG132, to prevent the degradation of E-cadherin and so enhance its detection. When using low-salt lysis buffer, we found that the levels of soluble cytosolic E-cadherin were still elevated 48 h after IFNγ-treatment and that this was substantially reduced by Fyn but not control siRNA treatment of the epithelia (Figure 5A). This contrasted with the detection of E-cadherin in T84 cell extracts produced by lysis with a high-salt, Triton X-100-based buffer, where only slight increases in E-cadherin protein were observed following IFNγ treatment relative to either controls or IFNγ+Fyn siRNA-treated epithelia (Figure 5A, lower panel). Consistent with the effects of Fyn, Hakai siRNA reduced the IFNγ-stimulated solubilisation of E-cadherin (Figure 5B). Again, there was only a modest increase in E-cadherin detected in Triton X-100, high salt lysis buffer-extracted lysates of IFNγ-treated T84 cells.

Figure 5C presents representative confocal immunofluorescence images showing membrane distribution of E-cadherin and occludin (images were collected at a consistent z-plane depth for E-cadherin and occludin, based on the first observable occludin immunoreactivity). Treatment with Fyn or Hakai siRNA reduced the loss of adherens junction-localized E-cadherin following 48 h of exposure to IFNγ. Conversely, IFNγ-stimulated disruption of tight junctions, indicated by dissociation of occludin, was neither affected by Fyn nor Hakai siRNA.

### T84 Cell Dissociation Stimulated Agitation of IFNγ- Treated Monolayer is Inhibited by Fyn and Hakai siRNA

**Figure 6:**
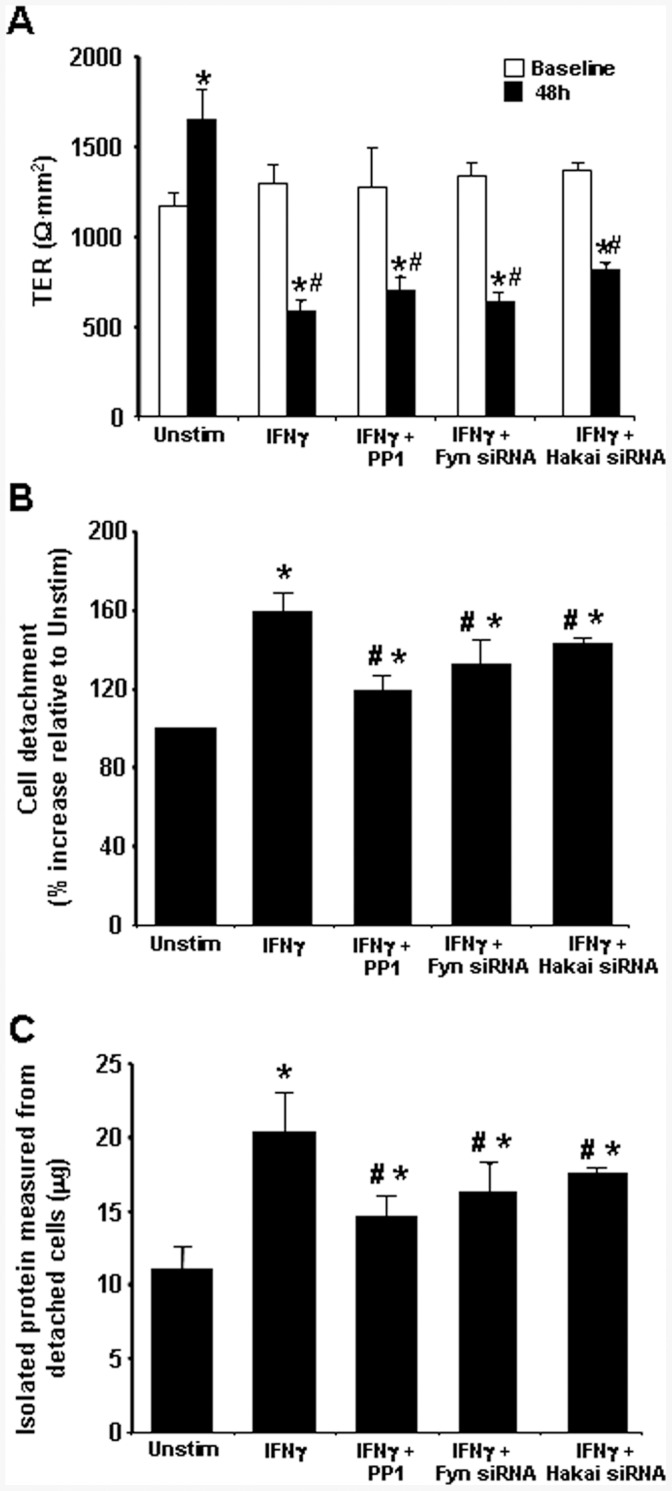
Increased cell detachment evoked by fluid shear in IFNγ-treated T84 epithelial cell monolayers is reduced by siRNA knock-down of Fyn kinase and Hakai. A) Electrically confluent T84 cell monolayers treated with IFNγ (10 ng/ml, 48 h, control siRNA) displayed the expected drop in transepithelial resistance (TER) that was unaffected by PP1 (125 nM) co-treatment or by siRNA knockdown of Fyn kinase or the E3-like ligase, Hakai. In contrast, cell detachment from the monolayer caused by gentle fluid shear stress evoked by IFNγ, and assessed by (B) cell counts and (C) total protein from suspended cells was statistically significantly reduced by PP1, and to a lesser extent by siRNA targeting Fyn kinase or Hakai (measurement of cell detachment as detected by amount of cellular protein from washed cells (mean±SEM; n = 4−6 monolayer preparations from 2 experiments; * and #, p<0.05 compared to control (Unstim) monolayers and IFNγ treatment (+ control siRNA), respectively; data are representative of three experiments).

A key functional measure of E-cadherin stability is the integrity of cell-cell contacts. The drop in TER that is consistently observed 48 h after IFNγ-treatment (measured before the gentle flushing) was unaffected by PP1 co-treatment or knock-down of Fyn kinase or Hakai with siRNA (Figure 6A), which is consistent with the inability of either treatment to prevent the disruption in the pattern of the tight-junction protein occludin by IFNγ (Figure 5C) and the inability of PP1 to prevent the subtle changes in ZO-1 distribution (Figure 1). However, while the monolayer remained intact it was more fragile as demonstrated by the substantial increase in cell detachment evoked by a gentle, consistent flushing with warm PBS which could mimic some aspects of shear stress or fluid transit along the intestine. As shown both by enumeration of detached cells (Figure 6B) and quantification of total protein in cells collected from the culture medium (Figure 6C), PPI inhibition of Src kinases significantly inhibited IFNγ-stimulated cell detachment, as did siRNA knock-down of Fyn kinase or Hakai in T84 epithelia.

## Discussion

The enteric epithelial layer is an important active participant in mucosal immunity through the secretion of anti-microbial peptides, promotion of oral tolerance and formation of a barrier to the entry of lumen-derived material [1], [26]. Epithelial injury can contribute to the exacerbation of inflammatory responses and affect the rate of restoration of homeostasis following infection, potentially leading to prolonged inflammation [1]. Indeed, disruption of the integrity of the gut epithelium (or its function) is associated with chronic inflammatory diseases, such as Crohn’s disease and ulcerative colitis [27]. In addition to the direct effects of long-term injury to the epithelium, chronic inflammation can contribute to the development of cancer, a relationship that is increasingly recognized in the context of inflammatory bowel diseases and colorectal cancer [28]. Thus, there is considerable value in understanding the full impact of inflammatory signals such as IFNγ on the control of enteric epithelial cell-cell contacts.

The molecular and cellular mechanisms that program the gut to remain in a chronic diseased state are poorly defined. IFNγ is an established immune effector molecule associated with intestinal inflammation in humans and animal models. Aside from its key function as an immune-stimulatory cytokine, *in vitro* study (and a lesser number of *in vivo* observations [3], [29]) indicate that IFNγ can significantly disrupt epithelial barrier function [30], thereby potentially exacerbating inflammation by facilitating a breach of the epithelial layer and entry of antigen and microbes into the mucosa. Therefore, IFNγ is of particular interest since elucidation of the signal transduction pathways that promote immune function as opposed to those which elicit alterations in cell-cell interactions and decrease epithelial barrier function may uncover unique targets for therapeutic intervention. Extensive research efforts are revealing the molecular assembly of the epithelial tight junction, the structure primarily responsible for restricting the movement of material between adjacent cells, and how pro-inflammatory cytokines, including IFNγ, affect the tight junction [26], [30], [31]. Comparatively little attention has been directed towards assessing cytokine regulation of the adherens junction. Positioned directly beneath the tight junction, the formation of the adherens junction is considered as a critical forerunner to development of the tight junction and hence epithelial monolayer formation.

Here, using a series of molecular analyses and the human T84 epithelial cell line (a model often used to define principles of the control of epithelial permeability [32]–[34]), we have confirmed that E-cadherin expression is affected by IFNγ [21] and provide evidence in support of a requirement for Fyn kinase in IFNγ-evoked loss of E-cadherin from the adherens junction. These data add to growing awareness that Src-kinase, including Fyn, activity affects the stability of E-cadherin at the cell surface: Src kinases promote the phosphorylation of E-cadherin at C-terminal residues associated with removal of E-cadherin from the cell membrane [35], [36]; Src has been shown to regulate p120-catenin binding to the juxtamembrane domain of E-cadherin, a critical cytoplasmic region that mediates E-cadherin membrane localization [36]; the E-cadherin binding partners, p120-catenin and beta-catenin, are phosphorylated by Src kinases [19]; and p120-catenin, a factor required for stable integration of E-cadherin into the cell membrane, is phosphorylated by Fyn [37]. In addition, phosphorylation of p120-catenin facilitates the association of E3-like ligase Hakai. Consistent with these findings, our data support a model whereby IFNγ causes removal of E-cadherin from the epithelial surface via the mobilization of Fyn kinase and subsequent targeting for degradation via the E3-like ligase, Hakai. Thus, these novel data highlight an additional mechanism through which epithelial cell-cell adhesion may be disrupted during ongoing inflammatory reactions involving IFNγ.

The impact of destabilization of the adherens junction on an epitheliums barrier function once tight junctions have formed is unclear [38]. Certainly TER is a direct reflection of the tight junction as perseverance of E-cadherin did not ablate the ability of IFNγ to reduce TER; however, E-cadherin does provide a level of cell-cell stability since its loss leads to a more friable monolayer. Structural components of the tight junction, such as claudins and occludin, cycle rapidly into and out-of the epithelial cell membrane and are anchored to the cytoskeleton via adaptor proteins, principally isoforms of zonula occludens (ZO) [39]. ZO-1 and the adherens junction proteins can interact and both play important roles in establishing cell polarity [40], which is essential for proper function of the enteric epithelium. For example, E-cadherin can physically interact with polarity-promoting and regulatory factors, such as the tumour suppressing phosphatase PTEN [41], [42] and Par3/Bazooka [43], respectively. Consequently reduced surface expression of E-cadherin may affect the maintenance of the epithelial tight junction, the ability of enterocytes to spread and heal a wound, and to restore a polarized monolayer with the ability to vectorially transport electrolytes.

An intriguing alternative possibility is that IFNγ-stimulation of E-cadherin internalization might have a protective function. For instance, the bacterial pathogen *Listeria monocytogenes* can use E-cadherin as a receptor for entry into the enterocyte and hence removal of E-cadherin from the adherens junction could limit *L. monocytogenes* invasion [44].

Src-family kinases possess oncogenic properties: Src-mediated destabilization of E-cadherin has been presented as a molecular mechanism for malignancy in tissues including the colonic epithelium [9], [16], [45] and emerging data suggest that Fyn kinase participates in the malignant transformation of prostate epithelium [13], [46]. Further, though unrelated to its ubiquitin ligase activity, Hakai may be considered an oncogenic factor via its ability to modify RNA splicing [47]. Also, Hakai, C-terminal fragments of E-cadherin, p120-catenin and beta-catenin have been localized to the nucleus in *in vitro* cell systems [47], [48]. So while focusing on IFNγ regulation of structural elements of the epithelial barrier we should not overlook IFNγ-Fyn-Hakai activity in the contexts of cell signalling, gene regulation and cancer. As the field of inflammation-driven cancer (e.g. colorectal cancer) gains momentum we speculate that IFNγ regulation of E-cadherin and associated signalling molecules, such as beta-catenin, is worthy of substantive investigation and has the potential to yield key insights into the regulation of the malignancy.

In conclusion, our data suggest the Src kinase Fyn acts as a pivotal signal in specific aspects of IFNγ control of epithelial function. Not only is it central to the regulation of macromolecular permeability [8], but via its affect on E-cadherin it can modify cell-cell interactions, intracellular signalling pathways and possibly also oncogenic processes as well. These effects contrast with the accepted role of IFNγ as an anti-cancer factor due to its activation of anti-tumour cell types, namely cytotoxic T cells, macrophages and natural killer cells [49]. Thus, we propose that Fyn kinase could be exploited to inhibit many of the pathological effects of IFNγ on intestinal epithelial cells, and possibly epithelia in general.
